# Novel Insights into the Physiological Function of the APP (Gene) Family and Its Proteolytic Fragments in Synaptic Plasticity

**DOI:** 10.3389/fnmol.2016.00161

**Published:** 2017-01-20

**Authors:** Susann Ludewig, Martin Korte

**Affiliations:** ^1^Division of Cellular Neurobiology, Zoological Institute, TU BraunschweigBraunschweig, Germany; ^2^Helmholtz Centre for Infection Research, AG NINDBraunschweig, Germany

**Keywords:** amyloid precursor protein, amyloid precursor-like protein, long-term potentiation, synaptic plasticity

## Abstract

The amyloid precursor protein (APP) is well known to be involved in the pathophysiology of Alzheimer's disease (AD) via its cleavage product amyloid ß (Aß). However, the physiological role of APP, its various proteolytic products and the amyloid precursor-like proteins 1 and 2 (APLP1/2) are still not fully clarified. Interestingly, it has been shown that learning and memory processes represented by functional and structural changes at synapses are altered in different APP and APLP1/2 mouse mutants. In addition, APP and its fragments are implicated in regulating synaptic strength further reinforcing their modulatory role at the synapse. While APLP2 and APP are functionally redundant, the exclusively CNS expressed APLP1, might have individual roles within the synaptic network. The proteolytic product of non-amyloidogenic APP processing, APPsα, emerged as a neurotrophic peptide that facilitates long-term potentiation (LTP) and restores impairments occurring with age. Interestingly, the newly discovered η-secretase cleavage product, An-α acts in the opposite direction, namely decreasing LTP. In this review we summarize recent findings with emphasis on the physiological role of the APP gene family and its proteolytic products on synaptic function and plasticity, especially during processes of hippocampal LTP. Therefore, we focus on literature that provide electrophysiological data by using different mutant mouse strains either lacking full-length or parts of the APP proteins or that utilized secretase inhibitors as well as secreted APP fragments.

## Introduction

The amyloid precursor protein (APP) gene is localized in humans on chromosome 21 and its expression gives rise to three major isoforms (APP695, APP751, APP770; around 170 kDa) generated via alternative splicing. APP695 is the predominant isoform in neurons (Robakis et al., 1987; Yoshikai et al., 1990). APP is translated in the endoplasmatic reticulum (ER) where it forms stable dimers which are transported through the secretory pathway via the Golgi apparatus to the cell surface (Isbert et al., 2012; Tan and Evin, 2012). APP is classified as a type I transmembrane glycoprotein with one membrane spanning domain, a large extracellular N-terminus and a small intracellular C-terminus (Dyrks et al., 1988). The mammal APP is part of a larger gene family including the homologs amyloid precursor-like proteins 1 and 2 (APLP1 and APLP2), both of which are expressed throughout the body nervous system (brain, spinal cord, retina), immune system (thymus, spleen), muscle (smooth, cardiac, and skeletal), kidney, lung, pancreas, prostate gland, and thyroid gland (Wasco et al., 1993; Liu et al., 2008; Aydin et al., 2012). Interestingly, the APP and APLP2 proteins are found at particularly high levels in the brain where their expression patterns largely overlap in pyramidal neurons of the cortex and hippocampus (Bendotti et al., 1988; Lorent et al., 1995). Thereby, the APP isoform APP_695_ is especially found in excitatory neurons as well as in GABAergic interneurons while the expression of the other two isoforms, 751 and 770, is assigned to other cell types (Wang et al., 2014; Hick et al., 2015). *In vitro* studies revealed APP expression in astrocytes and microglia that is increased following brain injury (LeBlanc et al., 1997; Rohan de Silva et al., 1997). On the other hand a more recent study reported that APP expression is restricted to neurons and cannot be found in major glial cells like astrocytes or microglia under basal as well as neuroinflammatory conditions (Guo et al., 2012). These contradictory results are possibly due to the lack of APP specific antibodies. The highly homologous APP family members differ only slightly in their peptide domain structure and hence are displaying a similar proteolytic processing. The relatively short intracellular part of the C-terminus of APP and related proteins contains a YENPTY peptide motif which was shown to promote clathrin mediated endocytosis, modulate Aβ generation, interfere with Ca^2+^ homeostasis, and interact with multiple kinases, and adapter proteins (Perez et al., 1999; Leissring et al., 2002; Ring et al., 2007; Jacobsen and Iverfeldt, 2009). The extracellular part of APP is composed of the large E2 and E1 domains containing interaction sites for multiple binding partners like F-spondin, LRP1, Nogo-66 receptor, Notch 2, Netrin, Alcadein, sorL1/LR11, and extracellular matrix components (Müller and Zheng, 2012). Additionally, the E1 domain could be demonstrated to be crucial for the homo- and heterodimerization of APP family members (Soba et al., 2005). Interestingly, the Aβ motive, which is highly conserved in mammals and zebrafish is unique for APP. The APLPs lack this sequence.

Although the structure of both APP and APLPs are well known, the precise cellular function of these proteins remains elusive. For instance, extensive posttranslational modifications and the various cleavage products of APP and APLP processing complicate precise investigations. Nevertheless, several studies assessed putative cellular functions of the APP family members during development and in the adult nervous system (Jacobsen and Iverfeldt, 2009). Certainly, one of the most intriguing discoveries in this respect is the involvement of APP and its cleavage products in processes of synaptic plasticity (Korte et al., 2012) at which activity patterns generated by experience are able to modify neuronal function and structure. These include activity-dependent alterations of the efficacy of synaptic transmission and changes in the structure and number of synaptic connections (for a review see Korte and Schmitz, 2016). Part of the pathophysiology of Alzheimer's disease (AD) is related to the malfunction of synapses (Selkoe, 2002) and the application of amyloid beta (Aß) oligomers has been shown to directly impair synaptic plasticity (Shankar et al., 2008). Despite a huge amount of data which looked at the pathophysiological role of Aß plaques, it is less clear what the physiological function of APP and its fragments (including Aß) might be. In addition to APP, it is also important to further the understanding of the putative physiological functions of the related APLP1 and APLP2 proteins and their cleavage products. In this review we concentrate on the role of APP, APLP1, APLP2, and their proteolytic fragments in processes of synaptic transmission and in particular synaptic plasticity under physiological conditions (Table 1).

**Table 1:** **Electrophysiological characteristics of the APP protein family members and their proteolytic domains**.

**FL-APP or fragment**	**Species/Methodic details**	**Electrophysiological relevant observations**	**References**
APPsα	Adult, male Sprague-Dawley rats	(1) Reduction of LTP in DG by up to 50% *in vivo*	Taylor et al., 2008
	Intrahippocampal infusion of	(2.1) Enhancement of LTP at the PP-DG by 11 nm rec APPsa *in vivo*	
	(1) Antibodies targeting endogenous APPsα	(2.2) Increase of NMDA-R EPSC amplitude at PP-DG by 0.03 nm APPsα *in vitro*	
	(2) Recombinant APPsα (0.3, 3, 11, 330, 1000, 3300 nm)	(3.1) Reduction of LTP in DG *in vivo* and	
	(3) α-Secretase inhibitor tapi-1 (500 nm)	(3.2) Reduction of tetanus-evoked NMDA-R currents in DG cells *in vitro*	
	Acute hippocampal slices of Sprague-Dawley rats (young = 3–6 months and aged = 24–27 months)	Increases NMDA-R activation in aged animals Rescues age-related LTP deficits No effect on basal synaptic transmission or glutamate release (PPF)	Moreno et al., 2015
	Exogenous, recombinant APPsα application (0.1–1–10 nm)	Dose-dependent increase of NMDA-R related I_SE_	
	Rat OHCs treated for up to 24 h with APPsα (0.03–0.1–1–10 nm)	1 nm appsα reduces NMDA toxicity Facilitation of LTP expression in aged animals by induction of plasticity-associated immediate early genes	Ryan et al., 2013
	Acute hippocampal slices of adult, APP/APLP2 conditional DKO mice	Rescue of impaired LTP	Hick et al., 2015
	Bath application of recombinant APPsα (10 nm)	No effect on basal synaptic transmission	
APPsß	Acute hippocampal slices of adult, APP/APLP2 conditional DKO mice	No rescue of impaired LTP	Hick et al., 2015
	Bath application of recombinant APPsß (50 nm)	No effect on basal synaptic transmission	
Aß1–15	Acute hippocampal slices of adult, c57bl6 mice	fM Aß1–15 enhances PTP and LTP	Lawrence et al., 2014
	Bath application of aß1-15 (50 fM, 50 pM)	pM Aß1–15 has no effect on PTP or LTP	
Aη-α	Acute hippocampal slices of adult Swiss-mice	Unaltered baseline synaptic transmission	Willem et al., 2015
	Bath application of recombinant aη-α	Significant reduction of hippocampal LTP *in vitro*	
Aη-ß	Acute hippocampal slices of adult Swiss-mice	Unaltered baseline synaptic transmission	Willem et al., 2015
		No effect on hippocampal LTP *in vitro*	
	Bath application of recombinant Aη-ß		
APP-JCasp domain (NH_2_ terminal region of APP)	Intracellular delivered to presynaptic terminals of acute hippocampal slices of adult WT and APP KO mice	Strong reduction in basal synaptic transmission in WT, not in APP KO	Fanutza et al., 2015
		Increases PPF and synaptic frequency facilitation in WT, not in APP KO	
		Reduction of the rate of vesicle depletion without affecting vesicle recycling	
AICD	Acute hippocampal slices of APPΔCT15/APLP2-DM: mice lacking the last 15 amino acids of APP including YENPTY motif and APLP2	Decreased potentiation during PTP and LTP Trend toward decreased L-LTP Increased basal synaptic transmission Unaltered PPF and STP	Klevanski et al., 2015
APP	Murine OHCs of P0 APP-KO mice	No difference in I/O characteristic	Weyer et al., 2014
		Unaltered short term plasticity	
APLP1	APLP1-deficient adult male mice	*In vivo* recording at PP-GC synapse:	Vnencak et al., 2015
		Enhanced excitatory transmission	
		Decreased paired pulse inhibition of population spikes (decreased network inhibition)	
		Unchanged STP and LTP	
APLP2	Acute hippocampal slices of young (1–2 months) and aged (10–12 months) APLP2 deficient mice	Unchanged input-output characteristics across ages to controls	Midthune et al., 2012
		Unaffected PPF remains	
		No alterations in LTP	
APP/APLP2	Acute hippocampal slices of conditional adult DKO mice	Pronounced deficit in induction and maintenance of LTP	Hick et al., 2015
		Impaired PPF	
		Unaltered basal synaptic transmission	
		Unchanged spontaneous synaptic mEPSCS in CA1	
		No differences in NMDA-r subunit composition	
	Acute hippocampal slices of 16–24 days old conventional DKO mice	Increased PPF and synaptic frequency facilitation Decreased mEPSCs frequency and increased MEPSC decay time	Fanutza et al., 2015
ADAM-10 (α-secretase)	(1) Acute hippocampal slices of adult, female, conditional adam-10 KO	(1) Unaltered basic synaptic transmission impaired short-term synaptic plasticity strongly impaired LTP	Prox et al., 2013
	(2) *In vivo* hippocampal recordings in adult male CKO mice	(2) Electrographic seizure in one of five mutants	

## Role of full-length APP proteins at the synapse

**Figure 1:**
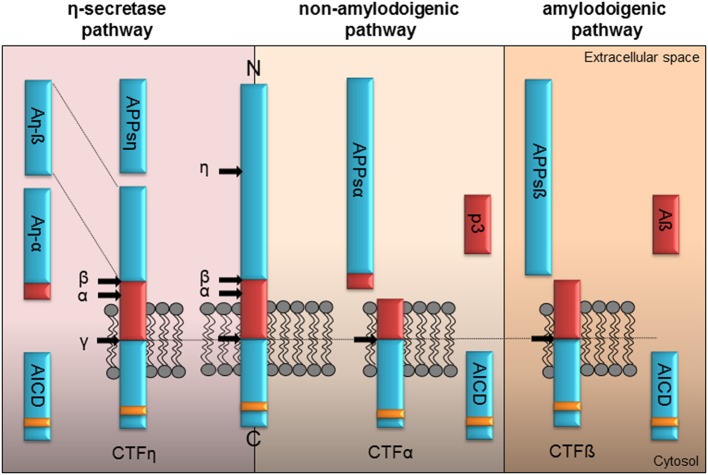
**Proteolytic processing of APP**. Full-length APP can be processed by α-, ß-, η-, and γ-secretases in three different pathways. The left panel illustrates the η-secretase processing of APP. Initially η-secretase cleavage releases the soluble APPsη, while CTFη remains embedded in the membrane. It is further processed by α- or ß-secretase at the extracellular side generating An-α or An-ß. Shedding of CTFη within the transmembrane domain by γ-secretase yields the APP intracellular domain (AICD) containing the highly conserved interaction motif (YENPTY, yellow box) or the short extracellular peptides Aß seen in the amyloidogenic or p3 within the non-amyloidogenic pathway. The non-amyloidogenic pathway depicted in the middle is driven by the α-secretase liberating APPsα in the extracellular space. Subsequently processing of membrane tethered CTFα by γ-secretase generates the p3 peptide and cytoplasmic AICD. The right panel illustrates APP processing in the amyloidogenic pathway by ß-secretase resulting initially in the release of the APPsß ectodomain. Following γ-secretase shedding of the membrane tethered CTFß the Aß peptide is secreted along with AICD in the cytoplasm.

**Figure 2:**
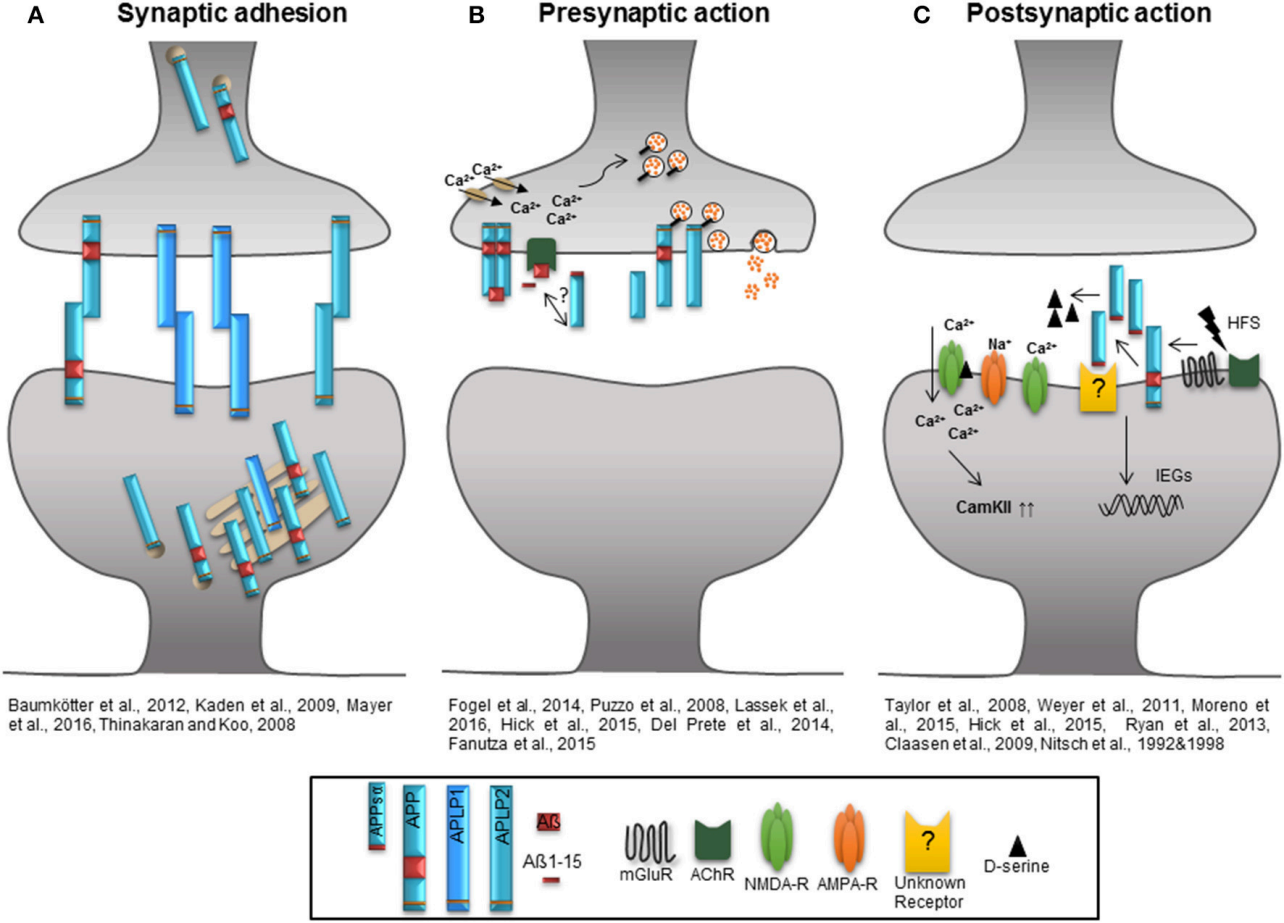
**Role of the APP protein family at the synapse. (A)** The extracellular domains of APP/APLPs mediate cell-cell adhesion in *trans* supporting synaptic connectivity. APP and APLP2 are mainly located in the Golgi apparatus and *trans* Golgi network. When integrated in the plasma membrane, APP and APLP2 show basal adhesive characteristics, while the proportion of plasma membrane APLP1 is higher and it's insertion dynamic. **(B)** Homodimerized APP might function as a cell-surface G-protein coupled receptor which is recognized by Aß and initiates signaling as well as neurotransmitter release by activation of calcium channels. Aß, Aß1-15, and potential also APPsα induce an AChR-dependent signal facilitating glutamate release via an increase in presynaptic calcium concentration. APP and APLP2 are mainly implicated in presynaptic function and their intracellular domains are associated with proteins of the synaptic vesicle release machinery regulating the vesicle content in the presynaptic active zone. **(C)** High frequency stimulation increases APP ectodomain shedding that might be linked to the activation of mGluRs or AChRs. High amounts of APPsα facilitate the function of NMDA-Rs by increasing the agonist D-serine or by induction of immediate early genes as well as signaling pathways like that of CamKII to support synaptic plasticity.

Gene targeting of APP protein family members provides a powerful tool to investigate the proteins functions. Studying adult APP and APLP2 single KOs in synaptic plasticity revealed only subtle phenotypes (von Koch et al., 1997) mainly due to the overlapping ubiquitous expression of the two proteins in mammals and their similar processing (see Figure 1). Under steady state conditions, the majority of full-length APP is located in the Golgi apparatus and in the trans-Golgi network (Thinakaran and Koo, 2008). When present at the plasma membrane APP and APLPs were shown to form homo- and heterotypic *cis* interactions and have been proposed to mediate cell–cell interactions in *trans* (Soba et al., 2005; Kaden et al., 2009; Baumkötter et al., 2012; Mayer et al., 2016). Synaptic adhesion by APP might not only be crucial to build and maintain synaptic contacts, but also to regulate synaptic plasticity (see Figure 2). Highest expression levels at the membrane were observed for APLP1 suggesting that it might be the family member with the upmost potential to mediate cell contacts (Kaden et al., 2009). Recently, the study of Mayer et al. (2016) identified APP and APLP2 to exhibit basal adhesive properties while APLP1 mediated neuronal adhesion is dynamic and regulated by zinc. Copper was instead shown to induce *cis*- and *trans*-dimerization of APP at its E1 domain (Baumkötter et al., 2014). Importantly enhanced *trans* or *cis* interaction of APPs or APLPs is accompanied by a reduction of ectodomain shedding of the proteins (Stahl et al., 2014; Mayer et al., 2016) and might therefore interfere with the ability to modulate synaptic function.

### APP-KO

The well-studied constitutive KO of APP in mice leads to an age-related deficit in synaptic plasticity, mainly in long-term potentiation (LTP, see Box 1 for definition). LTP reflects the increase in synaptic strength that lasts for at least 1 h and is paralleled by alterations at the contact sites between nerve cells, the presynapse (axonal boutons) and postsynapse (dendritic spines). No alterations in synaptic plasticity, the cellular correlate for learning and memory (Stuchlik, 2014) were found in young mice accompanied by normal basal synaptic transmission properties and short-term synaptic plasticity (STP) paralleling the intact behavioral learning of adult and impaired performance of aged mice (Seabrook et al., 1999; Ring et al., 2007; and reviewed by Turner et al., 2003; Korte et al., 2012). The age-dependent LTP defect is further supported by the electrophysiological measurements of murine organotypic hippocampal slice cultures (OHCs) from APP-KO pups prepared at postnatal day zero. No differences in the Input–Output characteristics and STP of APP-KO in comparison to wild-type OHCs were observed (Weyer et al., 2014). In agreement, the loss of APP does not impair synaptic plasticity in the adult organism and thereby APLP2 and maybe APLP1 are considered to perform redundant functions, but fail to compensate for APP deficiency with age.

Box 1 **Term definitions.**

**Synaptic plasticity** designates the activity-dependent alterations of the efficacy of synaptic transmission and changes in the structure as well as number of synaptic connections whereby activity patterns are generated by experience. Synaptic connections build the contact sites between nerve cells and alterations at these contact sites provide the basis to store memories and information within neuronal networks (Korte and Schmitz, 2016).

**LTP—Long-term potentiation** is defined as a persistent increase in synaptic strength lasting for at least 1 h (Bliss and Lomo, 1973). It consists of an induction phase, including processes that trigger the alterations leading to the changes in synaptic efficacy followed by the expression or maintenance phase of LTP. LTP can be divided in different types: LTP lasting from 1 to 3 h is independent of transcription and translation and named early or E-LTP; if it lasts longer than 3 h, it is generally dependent on altered gene expression and referred to as late LTP (L-LTP, Bliss and Collingridge, 1993; Kandel, 2001).

**LTD—Long-term depression** is the counterpart of LTP and therefore defined as a persistent reduction in synaptic strength. LTD prevents excessive synaptic activity (Korte and Schmitz, 2016).

**STP—Short term synaptic plasticity** is a form of synaptic plasticity that is NMDA-R dependent, but presynaptically expressed. It depends on the frequency of induction as well as subsequent activity and lasts from ms to min (Zucker and Regehr, 2002; Volianskis and Jensen, 2003).

**PPF—Paired-pulse facilitation** is a NMDA receptor-independent form of short-term plasticity and a typical presynaptic phenomenon. The facilitation is caused in the process of re-establishment of intracellular Ca^2+^ levels after repetitive Ca^2+^ influx into the presynaptic terminal. PPF can be investigated by applying two single stimuli spaced by a defined time interval. Depending on the length of the Inter-Stimulus-Interval and type of stimulus used the second signal is facilitated or depressed (Paired-pulse depression, PPD). At shorter ISIs of <20 ms PPD is observed whereas larger ISIs >20 ms lead to PPF (Zucker and Regehr, 2002).

**Spine density—**Spines are small membrane protrusions from dendrites often with a neck-head structure building the postsynaptic elements of glutamatergic synapses (Korte and Schmitz, 2016). Their density can therefore be seen as correlate of the amount of excitatory synapses and often represents functional changes in synaptic strength.

### APLP2-KO

The function of APLP2 in synaptic plasticity has also been addressed in detail since this protein shares the highest degree of sequence homology with APP within the gene family. Furthermore, the spatial and temporal expression pattern of APLP2 is highly reminiscent to that of APP (Wasco et al., 1993). APP and APLP2 are ubiquitously expressed in the nervous tissue and at the neuromuscular junction (NMJ, Slunt et al., 1994; Lorent et al., 1995) as well as in pyramidal and GABAergic neurons of the hippocampus and cortex (Wang et al., 2014; Hick et al., 2015). In contrast to APP-KO mice, young and aged APLP2 single KOs behave like wild-type mice showing no impairments in LTP, STP, PPF, or basal synaptic transmission (Weyer et al., 2011; Midthune et al., 2012). These observations go in line with normal learning and memory performance in cognitive tasks like the Morris-Water-Maze (MWM) or the passive avoidance test (Heber et al., 2000; Guo et al., 2012). The functional effects are consistent with investigations of dendritic spine numbers at excitatory neurons, reflecting the number of excitatory synapses. Whereas, the spine density assessed *in vivo* was affected in aged APP-KO animals, it was unaltered in APLP2-KO mice as well as in APLP2 OHCs *in vitro* (Lee et al., 2010; Midthune et al., 2012; Weyer et al., 2014). It seems likely that endogenous APP is able to compensate for the genetic ablation of APLP2 with age, while vice versa APLP2 is incapable to compensate the loss of APP in aged animals. This implicates that APP has either different or dominant neuronal functions compared to APLP2.

### APLP1-KO

Despite the generation and first characterization of the conventional APLP1-KO mouse in 2000 by Heber and colleagues, the function of this homolog has been less attended in synaptic plasticity Since APLP1 is the only APP family member with restricted expression to the brain (Lorent et al., 1995; Thinakaran and Koo, 2008; Klevanski et al., 2014), it is intriguing to speculate that APLP1 has a unique neuronal role and therefore might also be of particular importance for synaptic plasticity. However, Heber et al. (2000) described only minor (if any) distinct phenotypes of APLP1-KO. The ablation of the APLP1 gene function did not result in impaired cognitive behavioral performance in the MWM task but rather. However, during the behavioral paradigm it has been noted that depletion of APLP1 resulted in an improvement of acquisition learning. The *in vivo* analysis at the perforant path-granule cell synapse (PP-DG) in young adult mice (16–20 weeks old) revealed unaltered STP and LTP, associated to enhanced excitatory transmission (Vnencak et al., 2015). The authors argued that maybe a larger number of perforant path synapses or an increased synaptic strength in APLP1-deficient mice may cause this enhancement, but final clarification is missing. Furthermore, the paired-pulse-inhibition (PPD) paradigm of the population spike points toward decreased GABAergic network inhibition in APLP1-KOs, an effect observed also for other APP-KO models.

## Role of the APP protein family in synaptic inhibition

The hippocampus is comprised of 95% excitatory and 5% inhibitory neurons, both expressing the APP family proteins (Hick et al., 2015). It is well established that the GABAergic system is especially important during the induction of LTP (Bliss and Lomo, 1973) and that excitation and inhibition must be tightly balanced for a well-coordinated network. This notion is supported by the finding that the inhibition of GABA_A_ receptors facilitates LTP and leads to hyperexcitability causing epileptic seizures (Gustafsson and Wigström, 1988; Casasola et al., 2004). Hippocampal hyperactivity is a hallmark of neurological diseases like mild cognitive (MCI, Bakker et al., 2012) and AD (Palop et al., 2007). Several studies suggest that the hyperactivity is caused by APP overexpression (Born et al., 2014) while others assume Aß to be the trigger (Busche et al., 2008; Minkeviciene et al., 2009). The APP family proteins seem to be closely involved in regulating GABAergic transmission as both APLP1-KO and aged APP-KO mice exhibit reduced GABAergic mediated PPD responses (Seabrook et al., 1999; Vnencak et al., 2015) and in addition increased susceptibility for kainite-induced seizures (Steinbach et al., 1998). Moreover, supporting the role of APP within the GABAergic network are the chronic reduction of GABA_A_ receptors and the lowered number of GABA_B_ autoreceptors mediating PPD of inhibition in the absence of APP (Fitzjohn et al., 2000) as well as the identified interaction of APP with GABA_B_ receptors *in vitro* (Norstrom et al., 2010) as well as recently *in vivo* (Schwenk et al., 2016). Like in APP-KO, in mice expressing only the secreted APPsα on an APLP2 deficient background (APPsa-DM; Weyer et al., 2011), the neutralization of GABA_A_ receptors by picrotoxin rescues impaired LTP presumably due to a facilitation of postsynaptic depolarization. Moreover, while addressing oscillatory activity by recording local field potentials (LFPs) in the dorsal hippocampus revealed normal theta- and gamma-frequency bands the coupling of gamma amplitude to the theta phase was diminished in around 9 months old APP-KO mice (Zhang et al., 2016). This observation indicates the presence of alterations within the local inhibitory networks (Zhang et al., 2016) thereby preventing a coordinated neuronal communication. Investigations by Yang et al. (2009) yielded that deletion of APP in hippocampal neurons increased L-type voltage gated Ca^2+^ channel (LTCC) levels and function underlying an altered GABAergic STP. Likewise, a recent report implied APP possibly via the APPsα fragment to stabilize Ca^2+^ homeostasis by regulating inhibition of LTCCs (Hefter et al., 2016). Nevertheless, APLP1 deficiency causes no LTP deficit even though GABAergic inhibition is affected in APLP1-KO mice. The related proteins, APP and APLP2, might exhibit similar interactions at the presynaptic membrane and thus possibly compensate for the functional loss of APLP1 at the postsynaptic density (PSD) during LTP induction and maintenance.

## APP and APLP2 double KO

The high content of APP and APLP2 especially in pyramidal cells of the cortex and hippocampus (Lorent et al., 1995) and their localization at synaptic sites (Laßek et al., 2013) suggest a role in synaptic transmission and synaptic plasticity. To address the function of these redundantly expressed proteins, combined KO models are necessary. Unfortunately, APP and APLP2 double KO (DKO) mice die perinatally (von Koch et al., 1997; Heber et al., 2000) indicating an indispensable role for both these proteins during development. The lethal phenotype of these DKO mice is most likely due to important functions of APP and APLP2 at the NMJ (Wang et al., 2005; Weyer et al., 2011) and reviewed by Caldwell et al. (2013). Neuromuscular transmission is severely impaired due to a reduced amount of synaptic vesicles and their impaired release. While the *Knock-In* of APPsα in the APP/APLP2-DKO mouse (APPsα-DM) rescued the lethal phenotype it resulted in muscular weakness and severe alterations in NMJ morphology (Ring et al., 2007). While the above study indicated that at the NMJ of APP and APLP2 DKO mice most alterations are found presynaptically, the role of the APP family members and their fragments at synapses within the CNS still remained open. The conditional approach used by Hick et al. (2015) opened the possibility to address the function of APP and APLP2 in the CNS leaving the PNS unaffected. Crossing of APP^flox/flox^ on an APLP2 null background to NexCre-deleter mice generates viable double mutants (cDKO). In these mice the depletion of APP is initiated from embryonic stage 11.5 onwards in excitatory neurons of the forebrain, while APLP2 is constitutively not expressed allowing the investigation of neurodevelopmental effects. Young adult mice show a pronounced deficit in LTP induction and maintenance as well as impairments in PPF. Alterations during the initial phase of LTP, the so-called post-tetanic potentiation and also STP provided a hint toward an impaired presynaptic function. In contrast, the functionality of the postsynapse remained unaffected as basal synaptic transmission was unaltered (Hick et al., 2015). Another study using young conventional APP/APLP2 deficient mice (APP/APLP2-DKO, surviving escape mutants) described increased PPF and synaptic frequency facilitation (FF, Fanutza et al., 2015), supporting the assumption that APP and APLP2 are involved in presynaptic function.

## Presynaptic function of APP family proteins

Short-term plasticity (STP) depends on the release probability of synaptic vesicles, their recycling and content in the presynapse as well as on the activity of calcium sensor kinases. APP and APLP2 show a variety of possible interactions with the synaptic vesicle release machinery: Biochemical approaches showed that APP is associated with synaptic vesicle proteins (Del Prete et al., 2014; Laßek et al., 2014) and that it can be cleaved within vesicles by BACE-1 (Del Prete et al., 2014). Especially the intracellular regions of APP, APLP2, and CTF-ß have been shown to interact with presynaptic vesicle proteins like Rab, AP-2 subunits, the Ca^2+^ sensors synaptotagmins, clathrin, and complexin (Del Prete et al., 2014; Fanutza et al., 2015). Results from APP-KO animals point toward a role of APP in controlling synaptic vesicle protein content in the presynaptic active zone as synaptophysin, synaptotagmin-1, and SV2A protein levels are reduced in APP KO mice. In contrast, when beside APP also APLP1 or APLP2 are gene targeted, the abundance of synaptic vesicle proteins is increased (Laßek et al., 2014). The increase in SV2A and synaptotagmin-1 has also been observed in the conditional APP/APLP2 mutant mice generated by Hick et al. (2015) and recently analyzed (Laßek et al., 2016). In that study, Lassek and colleagues further show that APP deletion disturbs Ca^2+^ homeostasis, due to a misregulation of calmodulin and neuromodulin but not of the expression of CaMKII or Ca^2+^ channels. APLP1 is also localized at the presynaptic active zone (Laßek et al., 2016), but beside the function as mediator of neuronal adhesion (Kaden et al., 2009; Mayer et al., 2016) and its potential involvement in GABAergic neurotransmission (Vnencak et al., 2015) no other role or interaction partners have been attributed so far.

## Postsynaptic function of APP family proteins

In addition to a possible function at the presynapse in the developing and mature CNS, all APP family members have been suggested to play a role at the postsynapse. In particular an interaction with N-methyl-D-aspartate receptors (NMDA-R) has been shown especially for the GluN1/GluN2A and GluN1/GluN2B subunits (Cousins et al., 2015). APP, APLP1, and APLP2 are further involved in the regulation of the cell surface expression of NMDA-Rs thus controlling NMDA-R homeostasis (Cousins et al., 2015).

Addressing the role of APP and APLP2 at the postsynapse with the whole cell patch clamp method (measuring miniature excitatory postsynaptic currents (mEPSCs) yielded conflicting results. The study of Fanutza et al. (2015) using conventional APP/APLP2 double mutants, described a decreased mEPSC frequency and an increased mEPSC decay time leading to the assumption of redundant mediated function of APP and APLP2. In contrast, Hick et al. (2015) investigated a conditional APP/APLP2 KO (cDKO) and found no alterations in spontaneous synaptic mEPSCs and in their frequencies. Moreover, the analysis of the NMDA-R subunit composition further points toward unchanged postsynaptic transmission in the cDKO mice (Hick et al., 2015). In this context it is important to note that around 80% of the APLP2^−/−^APP^−/−^ mice die within the first weeks after birth and only 0.3% survive until weaning (von Koch et al., 1997; Heber et al., 2000). Therefore, the mice studied by Fanutza et al. (2015) were so called “escape-mutants” and their results need to be interpreted with care. It might be that the surviving conventional DKOs developed adaptation mechanisms e.g., an upregulation of synaptic proteins accounts for these controversial results. APLP1 is supposed to accumulate at the postsynapse (Kim et al., 1995) and was also shown to regulate NMDA-R content (Cousins et al., 2015). APLP1, like the other two family members contains the highly conserved YENPTY interaction motif and in thus able to initiate downstream signaling cascades in the postsynaptic compartment supporting synaptic plasticity (activation of intracellular signaling cascades and their contribution to synaptic plasticity is discussed below).

## Proteolytically generated peptides—APPsα, APPsß, Aß, AN-α, AN-ß

Gene targeting of APP family members using single and double mutants provided evidence about the possible involvement of these proteins in synaptic plasticity, but it could not answer the question of whether the observed effects arose from the action of the full-length proteins or from the absence of their secreted fragment(s).

Evidence pointing to a role of APP fragments in processes of synaptic plasticity arose from the observation that APP processing by α- and ß-secretase is activity-dependent (Nitsch et al., 1993; Fazeli et al., 1994; Kamenetz et al., 2003; Gakhar-Koppole et al., 2008) and can thus be potentiated by neuronal depolarization or high frequency stimulation (HFS). Consequently, the released domains may be especially involved during processes of synaptic activity.

Depending on their site of release, extra- and/or intracellularly, they might have functions as signaling molecules or initiate signaling by binding to different types of receptors. Proteolytic processing of APP is depicted in Figure 1 and was shown to be similar for APLP1 and APLP2 except for the release of Aß as its coding sequence is absent in the APP homologs (Eggert et al., 2004; Walsh et al., 2007). The current view allows differentiation between three different pathways initiated by the α-, ß-, or η-secretase (see Figure 1). In the non-amyloidogenic pathway the α-secretase cuts within the Aβ domain liberating the large APPsα ectodomain and a membrane-anchored C-terminal fragment α (CTF α). The latter is further cut by the γ-secretase releasing the p3 fragment extracellularly and the remaining APP intracellular domain (AICD) into the cytoplasm. The amyloidogenic processing by the ß-secretase yields the APPsß ectodomain and the membrane-tethered CTF ß. Afterwards the activity of the γ-secretase generates the AICD peptide along with Aß. Recently Willem et al. (2015) identified a η-secretase cleavage site in the extracellular domain of APP releasing a short extracellular APPsη ectodomain. Subsequent processing of the remaining membrane anchored CTF η by the α- or ß-secretase generates two new peptides, Aη-α and Aη-ß (Willem et al., 2015). Importantly, APP processing is not restricted to the plasma membrane, but was also shown to occur within synaptic vesicles (Del Prete et al., 2014).

## APPsα promotes synaptic plasticity

Numerous studies showed that the α-secretase released ectodomain APPsα exerts a role in neuroprotection, synaptic plasticity, and within neuronal networks (Ring et al., 2007; Weyer et al., 2011; Kögel et al., 2012). The acute synaptic function of endogenous APPsα in the adult brain was shown by using APP/APLP2 cDKO mice (Hick et al., 2015). One hour incubation with 10 nM recombinant APPsα peptide (recAPPsα) rescued the severe LTP deficit in acute slices of the mutants indicating that the soluble ectodomain acts on a rapid time-scale. These results were in line with previous findings of Taylor et al. (2008) reporting that intrahippocampal infusion of recAPPsα in the dentate gyrus (DG) of anesthetized rats enhances LTP recorded at the PP-DG pathway *in vivo*. Moreover, a recent study showed that recAPPsα is able to rescue age-dependent LTP deficits *in vitro* (Moreno et al., 2015). In addition, we showed that virus driven long-term expression of APPsα restores impaired synaptic plasticity in a mouse model of AD (Fol et al., 2016). It is by now not clear how APPsα mediates the rescue and which receptor might be activated. Overall there is good evidence for a prominent role of APPsα at the postsynapse, in particular by influencing NMDA-R function and synaptodendritic protein synthesis (Taylor et al., 2008; Claasen et al., 2009).

## Modulation of postsynaptic function by APPsα

One possible mechanism of APPsα action at synapses might be the facilitation of evoked NMDA-R currents at the postsynapse as shown in the study of Taylor et al. (2008). These results were confirmed by acute application of recAPPsα on acute slices of APP/APLP2 cDKO mice or aged rats restoring the LTP induction deficit and highlighting that APPsα modulates synaptic plasticity and regulates early events of the LTP processes (Hick et al., 2015; Moreno et al., 2015). Both studies further report that exogenous applied APPsα does not affect basal synaptic transmission or glutamate release. NMDA-Rs may stimulate α-secretase cleavage of APP during high-frequency stimulation (HFS) or HFS activates metabotropic glutamate (mGluRs) or muscarinic acetylcholine receptors (mAChRs) to promote APPsα release. Notably, the processing must be tightly regulated as high APPsα concentrations reduce LTP induction by activation of inhibitory signaling pathways (Taylor et al., 2008). The concentration dependent action of APPsα to increase NMDA-R currents could further be linked to D-serine availability at the synapse (Moreno et al., 2015). D-serine is the main co-agonist required for NMDA-R activation (for details see review Billard, 2012) and APPsα stimulates it's production and release. A recent study further showed that APP deficiency is linked to alterations in D-serine levels accompanied by impaired structural plasticity of dendritic spines (Zou et al., 2016). Facilitation of LTP expression by APPsα might also be mediated through the induction of a subset of plasticity-associated immediate early genes (Ryan et al., 2013), with *de novo* protein synthesis taking place in synaptoneurosomes mainly by activation of protein kinase G (Claasen et al., 2009). Among APPsα activated signaling cascades are furthermore the phosphatidylinositol-3-kinase (PI(3)K)-Akt kinase signaling pathway (Cheng et al., 2002; Milosch et al., 2014) and the mitogen-activated protein (MAP) kinase signaling pathway (Greenberg et al., 1995; Cheng et al., 2002).

Taken together, APPsα initiates several intracellular signaling cascades to support synaptic activity with an impact on NMDA-R currents, but still the APPsα-specific receptor triggering the effect on NMDA-Rs remains so far elusive. At least the experiments performed by Reinhard et al. (2013) could show that APPsα binding to a cell surface receptor involves two different subdomains. The N-terminal growth factor like domain (GFLD) of APPsα mediates the binding of protein and receptor, while the E2 domain interacts with membrane-anchored heparin sulfate proteoglycans (HSPG) and thus enhances the affinity to the APPsα-receptor. Among the potential receptors for which an interaction with the APP ectodomain is suggested are the low-density lipoprotein receptor-related protein (LRP1, Hoffmann et al., 1999; Goto and Tanzi, 2002), the sortilin-related receptor SORLA (Andersen et al., 2006; Hartl et al., 2013), Nogo-66 (Park et al., 2006), and the p75 neurotrophin receptor (Hasebe et al., 2013).

## Inhibition of APPsα mediated functions

In-line with the results following exogenous application of APPsα on LTP *in vitro* and *in vivo* are the opposite effects observed after α-secretase inhibition (which leads to a reduction in APPsα production). The conditional KO of the major α-secretase ADAM-10 resulted in strongly impaired LTP and altered STP (Prox et al., 2013). Within this study no differences in basic synaptic transmission were found. Interestingly, hippocampal network activity recorded *in vivo* in the CA1 region of the hippocampus of ADAM-10 cDKO mice was severely impaired and 20% of the animals showed electrographic seizures (Prox et al., 2013). A modulatory role for APPsα on network activity in the hippocampus and cortex has further been observed with regard to aging by Sánchez-Alavez et al. (2007) which recorded electroencephalographic activity. In addition, the key role of APPsα and APLP2sα for LTP induction and maintenance was shown by experiments using the ADAM-10 inhibitor in OHCs (Weyer et al., 2011) or by *in vivo* LTP recordings in the dentate gyrus after infusion of the α-secretase inhibitor TAPI-1 (Taylor et al., 2008). Due to the lack of ADAM-10 or its inhibition, APP processing by the ß-secretase is favored resulting in higher amounts of Aß peptides and APPsß which may further impair LTP, especially at nano- to micromolar levels see review Wang H. et al. (2012).

## APPsß does not modulate synaptic function

Only a few studies addressed the physiological action of the ß-secretase which leads to the release of the ectodomain APPsß (see Figure 1). APPsß is only 16 amino acids shorter than APPsα, but it is not as neuroprotective as APPsα. This was demonstrated by the *Knock-In* of the two soluble domains in the perinatal APP/APLP2 DKO mutant model. Only APPsα^+/+^APLP2^−/−^, but not APPsß^+/+^APLP2^−/−^ mice were viable (Li et al., 2010; Weyer et al., 2011). With regard to synaptic plasticity, APPsß cannot restore the LTP defect of APP/APLP2 cDKO mice (Hick et al., 2015) and does not facilitate LTP recorded *in vivo* within the DG of rats (Taylor et al., 2008). APPsß was further shown to have no influence on synaptic protein synthesis (Claasen et al., 2009). Consistent with the functional readout on synapses, Tyan et al. (2012) showed that only APPsα but not APPsβ partially rescued defects in dendritic spine number and morphology of primary hippocampal neurons from APP-KO mice.

## Aß dominantly acts at the presynapse

At physiological, picomolar concentrations Aß was shown to modulate presynaptic vesicle release (Puzzo et al., 2008; Abramov et al., 2009; Wang H. et al., 2012). It functions via binding to presynaptic APP homodimers (Fogel et al., 2014) or by activating α7-nAChRs (Tong et al., 2011). The study by Lawrence et al. (2014) highlighted that the N-terminal domain of Aß contains this agonist-like activity of the Aß peptide. It was further suggested that successive α- and ß-secretase activity will release the short functional domain, named Aß1–15 (or Aß1–16, Portelius et al., 2011). With regard to synaptic plasticity, Aß1–15 significantly enhances PTP and LTP without altering baseline synaptic transmission at femtomolar concentrations, while higher amounts had no effect on hippocampal LTP (Lawrence et al., 2014). During LTD, Aß was shown to have a facilitating role through mGluR and NMDA-R due to the altered glutamate recycling at synapses (Li et al., 2009; Chen et al., 2013). The pathological effects of Aß, especially Aß42, are discussed in detail elsewhere (Mucke and Selkoe, 2012; Wang H. et al., 2012; Ripoli et al., 2014; Salgado-Puga and Pena-Ortega, 2015) We only want to mention that under pathological conditions Aß has the opposite effects on synaptic plasticity: it facilitates LTD, depresses LTP, causes dendritic spine loss and leads to hippocampal hyperactivity (Selkoe, 2002; Busche et al., 2008; Shankar et al., 2008; Mucke and Selkoe, 2012; Fol et al., 2016).

## An-α and An-ß, the new players in the field

The recently identified η-secretase releases a short extracellular APP-η ectodomain (Willem et al., 2015). The CTFη cleavage product remains anchored to the plasma membrane and subsequently is further processed by α- or ß- secretases to produce two small peptides, Aη-α and Aη-ß (see Figure 1; Willem et al., 2015). Willem and colleagues assessed the synaptic function of these peptides by measuring LTP *in vitro*. While both peptides had no influence on baseline synaptic transmission, hippocampal LTP was severely impaired by Aη-α but not by Aη-ß. The only structural difference between the two molecules is a C-terminal elongation of the Aη-α peptide by 16 additional amino acids (Figure 1). Interestingly, the same 16 amino acids are also present at the C-terminus of the APPsα fragment and, similar to Aη-ß, are lacking in the truncated APPsß form (Figure 1). This short peptide sequence contains a predicted neuroprotective domain and a heparin binding site (Furukawa et al., 1996). Indeed, neuroprotective properties have been reported for the APPsα peptide. However, and in contradiction to a favorable cellular function of this amino acid sequence, it has been found that Aη-α mediates neurotoxic effects (Willem et al., 2015). The adverse action of Aη-α was also observed by *in vivo* Ca^2+^ imaging experiments performed in the study of Willem et al. (2015) in which Aη-α strongly suppressed the activity of hippocampal neurons. In line with these findings are the observations for both ß-derived peptides. It seems unlikely that these fragments are involved in synaptic plasticity since both Aη-ß and APPsß lacked any modulatory effects on synaptic transmission when bath-applied to acute-hippocampal slices of APP/APLP2 cDKO mice at CA3-CA1 synapses (Hick et al., 2015) or when added during mossy fiber LTP recordings (Taylor et al., 2008). The different modes of action might be a consequence of a conformational change caused by the 16 additional amino acids at the carboxy-terminus of the Aη-α/APPsα cleavage products and/or by specific post-translational modifications (PTMs) like glycosylation or phosphorylation (Walter and Haass, 2000). In the study of Willem et al. (2015) Aη-α conditioned medium or 100 nM synthetic Aη-α showed a reduction in LTP, while only lower concentrations of 1–11 nM recombinant APPsα increased LTP. Moreover, the application of higher APPsα amounts had no effect or resulted even in reduced LTP (Taylor et al., 2008; Hick et al., 2015; Moreno et al., 2015). It would be interesting to know if APPsα can additionally be cleaved by η-secretase and if the released Aη-α could act as a co-player for Aß or APPsα and would therefore provide a modulatory mechanism.

## Knock-in of APPsα, APPsß, and the APP intracellular domain (AICD)

Beside the acute application of APP functional domains as peptides, gene targeting allows their re-introduction on APP or APLP2 null backgrounds. These conditional approaches or Knock-In (KI) mice opened the possibility of the functional characterization of the APP/APLP proteins during development as the constitutive triple KO and nearly all DKOs are embryonic lethal (von Koch et al., 1997; Heber et al., 2000). The study of Ring et al. (2007) analyzed the role of two APP functional domains by generating C-terminally truncated KI alleles of APP. APPsα-KI mice produce only APPsα, whereas APPΔCT15-KI mice lack the last 15 amino acids, including the highly conserved YENPTY motif. The phenotypes of both KI lines were similar to WT littermates. LTP as well as learning and memory assessed in behavioral tasks were normal presumably due to the constitutive expression of APLP2. The subsequent combination of both KI mice with APLP2 null mutants generated partially viable offsprings, whereas APPsβ-DM mice die (Li et al., 2010). APPsα-DMs were characterized in detail by Weyer et al. (2011) and APPΔCT15-DMs in the study of Klevanski et al. (2015). Both DM strains display alterations at PNS and CNS synapses. The mice suffer of muscular weakness due to altered morphology of the NMJ synapse and impaired transmitter release. Still, the APPsα-DMs reveal more severe electrophysiological impairments at the NMJ by additional reduced quantal content and alterations in the frequency of miniature endplate potentials (MEPP) compared to single mutants investigated by Ring et al. (2007). Hence different motifs account for a normal physiological function in the DMs. With regard to the CNS, both DMs are an impaired induction and maintenance of LTP paralleled by severely altered hippocampus-dependent behavior. STP between CA3/CA1 pyramidal cells was unchanged, while only APPΔCT15-DMs have altered postsynaptic properties and a trend toward defective protein-synthesis dependent Late-LTP.

### AICD is crucial at both sites of the synapse

The sole expression of AICD on an APP/APLP2 deficient background revealed alterations in synaptic plasticity. This might be a consequence of the abolished interaction of the intracellular domain with several adaptor proteins (Klevanski et al., 2015). For instance, APP interaction partners like Dab1, Shc, Grb, and Mint/X11 proteins mediate not only clathrin-mediated endocytosis of APP, but are also involved in the translocation of APP to the cell-surface (Aydin et al., 2012; van der Kant and Goldstein, 2015). Of particular importance might be the interaction with the adapter protein family FE65.I Interestingly FE65/FE65L1double deficient mice show a similar phenotype of cortical dysplasia as APP triple KO animals (Guénette et al., 2006). The FE65 proteins co-localize with APP in the ER and Golgi and facilitate the translocation of the precursor protein to the cell surface (Sabo et al., 1999). In addition, these proteins also regulate the shuttling of a multimeric complex of AICD/FE65/Tip60 into the nucleus to regulate gene transcription (Cao and Südhof, 2001). Long-lasting strengthening of synaptic transmission is impaired in APPΔCT15-DMs perhaps by impaired FE65/AICD mediated postsynaptic transcriptional activity (Klevanski et al., 2015). Interestingly, the analysis of FE65-KO, FE65L1-KO, and FE65/FE65L1-DKO mice revealed similar CNS phenotypes with impairments in LTP and dysfunctions in hippocampal learning tasks in double transgenic animals (Strecker et al., 2016). Accordingly, the APP-FE65 interaction might be crucial for synaptic function, but also for precise ectodomain shedding. In APPΔCT15-DMs mice, processing of APP via the amyloidogenic pathway is heavily impaired (Klevanski et al., 2015). That might have a positive effect with regard to Aß accumulation but also a negative outcome since picomolar amounts of Aß positively regulate the presynaptic vesicle release probability and facilitate learning and LTP in the hippocampal CA1 region by activating α7-nAChRs (reviewed by Wang H. et al., 2012). Collectively, these studies highlight an essential function for the 15 C-terminal amino acids including the YENPTY motif for transmembrane signaling and the ectodomain APPsα for proper synapse function.

## Conclusion

The majority of experimental data provided so far indicate a requirement for APP and APLP2 in synaptic plasticity which is in particular mediated by their proteolytic derived domains. The diverse functions of the APP protein family during either pre- or postsynaptically initiated processes of synaptic plasticity and under basal conditions are summarized in Figure 2. According to this model, APP full length proteins mediate stability of synaptic structures by their cell adhesion properties when integrated into the plasma membrane (Kaden et al., 2009; Baumkötter et al., 2012) and thus maintain appropriate spine numbers, especially via the APPsα domain (Tyan et al., 2012; Weyer et al., 2014). The insertion of full-length proteins is regulated by electrical activity or gradients of ions like zinc. The APLP1 protein shows the highest presence at the cell surface among all APP protein family members (Kaden et al., 2009; Mayer et al., 2016). As indicated Figure 2B depicts the APP protein family function at the presynaptic site, where the Aß, Aß-15 and possibly the APPsα domain interfere with glutamate release by activating nAChRs and enhancing intracellular Ca^2+^ levels (Puzzo et al., 2008; Wang Z. et al., 2012; Lawrence et al., 2014). It is further hypothesized that homodimerized APP acts as a G-Protein coupled receptor which is activated by Aß and might be involved in neurotransmitter release following enhanced Ca^2+^ influx. Especially the intracellular domains of APP and APLP2 seem to be associated with proteins of the synaptic vesicle release machinery regulating the molecular composition of synaptic vesicles at the presynaptic active zone (Del Prete et al., 2014; Fanutza et al., 2015; Laßek et al., 2015). At the postsynaptic compartment (Figure 2C) patterns of synaptic activity modulate APP family protein processing. HFS enhances the amount of secreted APPsα possibly linked to mGluRs or AChRs activation (Nitsch et al., 1992, 1997). Released APPsα was shown to facilitate NMDA-R currents (Taylor et al., 2008; Weyer et al., 2011) by increasing the NMDA-R agonist D-serine (Moreno et al., 2015) or by up-regulating signaling cascades downstream of NMDA-Rs- promoting synaptic plasticity like the CamKII pathway (Claasen et al., 2009) or by inducing the expression of immediate early genes involved in synaptic plasticity (Ryan et al., 2013). In this regard only APPsα was shown to have trophic functions while APPsß mediates neither positive nor negative effects with respect to baseline synaptic function or synaptic plasticity (Taylor et al., 2008; Hick et al., 2015). Several lines of evidence indicate that under physiological conditions structural and functional synaptic modulation is mediated by APPsα. What still needs to be investigated, however, is the mechanism by which APPsα exerts its trophic action, particularly which receptor might be activated and if the recently discovered Aη peptides might function as regulators of APPsα mediated synaptic plasticity and homeostasis. Identifying the cellular site of η-secretase cleavage within neurons and answering whether the secretion of Aη peptides is linked to neuronal activity will reveal the roles of the peptides in processes of synaptic plasticity.

Overall elucidating the physiological function of APP family members and fragments is an important step to understand brain function as well as brain dysfunction, also with respect to a possible treatment of neurodegenerative disorders like AD. It is important to acknowledge, that rational therapeutic approaches need to take into account the functional role of disease associated proteins.

## Author contributions

SL: wrote the review and prepared the figures. MK: designed the review and wrote the paper.

## Funding

This work was supported by the Deutsche Forschungsgemeinschaft Grants (KO 1674/3-1, 3-2) to MK.

### Conflict of interest statement

The authors declare that the research was conducted in the absence of any commercial or financial relationships that could be construed as a potential conflict of interest.
